# Supplementary material for: Horizontal gene transfer and recombination analysis of SARS-CoV-2 genes helps discover its close relatives and shed light on its origin
Source: BMC Ecol Evol. 2021 Jan 21;21:5. doi: 10.1186/s12862-020-01732-2 (PMC7817968; doi:10.1186/s12862-020-01732-2)
Supplement: Supplementary file 1 — Additional file 1: Table 1. The full virus names, abbreviations, host species and GenBank/GISAID accession numbers for all genomes analysed in our study. Table 2. The most appropriate evolutionary model found by the MegaX program for each gene/genome region analysed in our study, with the corresponding optimal parameters Gamma (G) and Intensity (I), as well as the values of Bayesian Information Criterion (BIC). [file 12862_2020_1732_MOESM1_ESM.pdf]

Additional Table 1 The full virus names, abbreviations, host species and GenBank/GISAID accession numbers for all genomes analysed in our study.

Organism name	Abbreviation	Host	Accession number (GenBank, GISAID)
hCoV-19/Australia/VIC231/2020	Hu Australia VIC231 2020	Human	EPI_ISL_419926
hCoV-19/USA/UT-00346/2020	Hu USA UT 00346 20202	Human	EPI_ISL_420819
BetaCoV/Wuhan-Hu-1	Hu Wuhan 2020	Human	NC_045512.2
hCoV-19/Italy/TE4836/2020	Hu Italy TE4836 2020	Human	EPI_ISL_418260
Bat coronavirus RaTG13	RaTG13	Bat	MN996532.1
hCoV-19/pangolin/Guangdong/1/2019	Guangdong Pangolin 1 2019	Pangolin	EPI_ISL_410721
hCoV-19/pangolin/Guangdong/P2S/2019	Guangdong Pangolin P2S 2019	Pangolin	EPI_ISL_410544
PCoV_GX-P5E	Guangxi Pangolin P5E	Pangolin	MT040336
PCoV_GX-P2V	Guangxi Pangolin P2V	Pangolin	MT072864
PCoV_GX-P5L	Guangxi Pangolin P5L	Pangolin	MT040335
PCoV_GX-P1E	Guangxi Pangolin P1E	Pangolin	MT040334.1
PCoV_GX-P4L	Guangxi Pangolin P4L	Pangolin	MT040333
bat-SL-CoVZC45	Bat CoVZC45	Bat	MG772933.1
bat-SL-CoVZXC21	Bat CoVZXC21	Bat	MG772934.1
BtCoV/273/2005	BtCoV 273 2005	Bat	DQ648856.1
Bat SARS coronavirus Rf1	Rf1	Bat	DQ412042.1
Bat SARS coronavirus HKU3-12	HKU3-12	Bat	GQ153547.1
Bat SARS coronavirus HKU3-6	HKU3-6	Bat	GQ153541.1
BtCoV/279/2005	BtCoV 279 2005	Bat	DQ648857.1
SARS coronavirus BJ01	SARS	Human	AY278488.2

SARS coronavirus	Tor2	Human	NC_004718.3
SARS coronavirus BJ182-4	SARS-CoV BJ182-4	Human	EU371562
Bat SARS-like coronavirus Rs3367	Rs3367	Bat	KC881006.1
SARS-related coronavirus BtKY72	BtKY72	Bat	KY352407.1
Bat coronavirus BM48-31/BGR/2008	BM48 31 BGR 2008	Bat	GU190215.1
Human betacoronavirus 2c EMC/2012	MERS-CoV S	Human	JX869059
Bat coronavirus HKU5-1	Bat HKU5-1	Bat	NC_009020
Bat coronavirus HKU4-1	Bat HKU4-1	Bat	NC_009019
Feline infectious peritonitis virus	Feline per	Cat	NC_002306
Human coronavirus HKU1	HKU1	Human	NC_006577
Human coronavirus HKU1 (isolate N2)	HKU1 N2	Human	Q14EB0
Human coronavirus HKU1 (isolate N1)	HKU1 N1	Human	5GNB_A
Murine coronavirus RA59/R13	Murine RA59/R13	Mouse	ACN89689
Murine hepatitis virus strain 4	Murine hep 4	Mouse	P22432
Murine hepatitis virus strain JHM	Murine JHM	Mouse	NC_006852
Mouse hepatitis virus strain MHV-A59 C12 mutant	Murine A59	Mouse	NC_001846
Murine hepatitis virus	Murine virus	Mouse	ABS87264
Rat coronavirus Parker	Rat Parker	Rat	NC_012936
Rabbit coronavirus HKU14	Rabbit HKU14	Rabbit	NC_017083
Equine coronavirus	Equine NC99	Horse	NC_010327
Porcine hemagglutinating encephalomyelitis virus	Porcine v	Pig	NC_007732
Human coronavirus OC43	Human OC43	Human	NC_005147
Human enteric coronavirus strain 4408	Human ent 4408	Human	NC_012950
Bovine coronavirus	Bovine CoV	Calf	NC_003045
Bovine respiratory coronavirus AH187	Bovine AH187	Calf	NC_012948
Bovine respiratory coronavirus bovine/US/OH-440-TC/1996	Bovine OH440	Calf	NC_012949

Additional Table 2. The most appropriate evolutionary model found by the MegaX program for each gene/genome region analysed in our study, with the corresponding optimal parameters Gamma (G) and Intensity (I), as well as the values of Bayesian Information Criterion (BIC). For the RB domain, amino acid sequences were analysed.

Region	Evolutionary model	G	I	BIC
Gene ORF1ab	GTR	1.24	0.46	236358.279
RB domain (25 species)	WAG	0.41	-	3861.345
Gene S	GTR	1.5	0.37	53988.111
Gene ORF3a	HKY	0.47	-	10517.624
Gene E	HKY	0.05	-	1637.944
Gene M	TN93	0.24	-	6488.936
Gene ORF6	HKY	0.7	-	2384.256
Gene ORF7a	HKY	0.48	-	4617.462
Gene ORF7b	HKY	-	0.31	1761.699
Gene ORF8	HKY	0.96	0.05	4966.318
Gene N	GTR	1.24	0.38	11781.432
Gene ORF10	JC	0.16	-	984.213
Whole genome	GTR	1.22	0.43	346054.493
RB domain (46 species)	WAG	3.15	-	12067.696
